# Granulocytic myeloid-derived suppressor cells increase infection risk *via* the IDO/IL-10 pathway in patients with hepatitis B virus-related liver failure

**DOI:** 10.3389/fimmu.2022.966514

**Published:** 2023-01-04

**Authors:** Xueping Yu, Jian Sun, Feifei Yang, Richeng Mao, Zhiqing Shen, Lan Ren, Songhua Yuan, Qian He, Linxia Zhang, Yu Yang, Xiangqing Ding, Yongquan He, Haoxiang Zhu, Zhongliang Shen, Mengqi Zhu, Chao Qiu, Zhijun Su, Jiming Zhang

**Affiliations:** ^1^ Department of Infectious Diseases, Shanghai Key Laboratory of Infectious Diseases and Biosafety Emergency Response, Shanghai Institute of Infectious Diseases and Biosecurity, National Medical Center for Infectious Diseases, Huashan Hospital, Fudan University, Shanghai, China; ^2^ Department of Infection Diseases, Fujian Medical University Affiliated First Quanzhou Hospital, Quanzhou, Fujian, China; ^3^ Department of Cardiology, The People’s Hospital of Fujian Traditional Medical University, Fuzhou, Fujian, China; ^4^ Shanghai Public Health Clinical Center and Institutes of Biomedical Science, Shanghai Medical College, Fudan University, Shanghai, China; ^5^ Key Laboratory of Medical Molecular Virology (MOE/MOH), Shanghai Medical College, Fudan University, Shanghai, China; ^6^ Department of Infectious Diseases, Jing’An Branch of Huashan Hospital, Fudan University, Shanghai, China

**Keywords:** hepatitis B virus, acute-on-chronic liver failure, granulocytic myeloid-derived suppressor cells, T cells, indoleamine 2, 3-dioxygenase

## Abstract

Hepatitis B virus-related acute-on-chronic liver failure (HBV-ACLF) results in high susceptibility to infection. Although granulocytic myeloid-derived suppressor cells (gMDSC) are elevated in patients with HBV-ACLF, their role in HBV-ACLF pathogenesis is unknown. To elucidate the mechanism of gMDSC expansion and susceptibility to infection in HBV-ACLF patients, we analyzed the proportion of gMDSC in the peripheral blood and organ tissues of patients with HBV-ACLF and an ACLF mouse model established by continuous injection (eight times) of Concanavalin by flow cytometry and immunohistochemistry. We found that the proportion of gMDSC increased significantly in the blood and liver of patients with HBV-ACLF. This increase was positively correlated with disease severity, prognosis, and infection. gMDSC percentages were higher in peripheral blood, liver, spleen, and bone marrow than control levels in the ACLF mouse model. Immunofluorescence revealed that the gMDSC count increased in the liver of patients with HBV-ACLF as well as in the liver and spleen of ACLF mice. We further exposed peripheral blood monocyte cells from healthy donors to plasma from HBV-ACLF patients, recombinant cytokines, or their inhibitor, and found that TNF-α led to gMDSC expansion and significant upregulation of indoleamine 2, 3-dioxygenase (IDO), while blocking TNF-α signaling decreased gMDSC. Moreover, we detected proliferation and cytokine secretion of T lymphocytes when purified gMDSC was co-cultured with Pan T cells or IDO inhibitor and found that TNF-α-induced gMDSC inhibited T cell proliferation and interferon-γ production through the IDO signaling pathway. Lastly, the ability of gMDSC to phagocytose bacteria was low in patients with HBV-ACLF. Our findings elucidate HBV-ACLF pathogenesis and provide potential therapeutic targets.

## Introduction

Acute-on-chronic liver failure (ACLF) is characterized by deep jaundice, coagulation dysfunction, and extrahepatic organ failure (1). In China, hepatitis B virus (HBV) infection accounts for approximately 87% of ACLF cases (2). Further infection from other pathogens is extremely common among ACLF patients; 81.2% develop bacterial or fungal infections during hospitalization, and 26.6% develop secondary infections. Infection significantly increases 90-d mortality in patients with ACLF. However, the exact mechanism of infection has not been fully elucidated.

Recent studies have shown that elevated immune checkpoint molecules and immunoregulatory cells may increase susceptibility to infection. For example, a marked expansion of MERTK-expressing monocytes and macrophages has been observed in ACLF patients, along with an increased number of CTLA-4^+^CD4^+^T cells; these changes suppress host immune response to microbes (3, 4). Additionally, immunosuppressive monocytic myeloid-derived suppressor cells (mMDSC) expanded in patients with ACLF, attenuating antimicrobial innate immune responses and impairing bacterial uptake and clearance (5). These outcomes are probably related to the downregulation of TLR-3 signaling that then impairs the TLR-driven innate immune response. *In vitro* studies have shown that the TLR-3 agonist polyI:C significantly reduced the proportion of mMDSC while simultaneously enhancing their phagocytic capacity in ACLF (5).

MDSCs are a heterogeneous population of immature myeloid precursor cells, classified as mMDSC or granulocytic MDSCs (gMDSC) based on phenotypic and functional characteristics (6). Granulocytic MDSC suppress innate and adaptive immune responses through depleting L-arginine and generating IL-10 (7, 8). Accumulating evidence indicates that gMDSC play an important role in the development of microbial infections, autoimmune disorders, and cancer. The gMDSC population is elevated in patients with alcoholic liver disease and closely associated with disease progression (9). However, we know little regarding whether gMDSC have impaired antimicrobial response or phagocytic capacity in patients with HBV-ACLF.

This study thus analyzed gMDSC proportion and phenotypes to understand their relationship with infection complications, severity, and prognosis. We also explored the mechanisms underlying gMDSC regulation of antimicrobial response and pathogen-clearing ability in patients with HBV-ACLF.

## Materials and methods

### Patients and sampling

From April 2016 to December 2017, we consecutively recruited 152 patients with CHB and 82 patients with HBV-ACLF within 24 h of admission to the Huashan Hospital and the First Hospital of Quanzhou. Healthy individuals without apparent disease (n = 40) were enrolled from Huashan Hospital as healthy controls (HC). Patients with CHB fulfilled the following established diagnostic criteria: positive hepatitis B surface antigen (HBsAg) detection for over 6 months, hepatitis-related clinical manifestations, histological confirmation of hepatitis, and abnormal ALT levels (≥40 U/L) (10). Criteria for HBV-ACLF patients were as follows: history of CHB or liver cirrhosis, serum TBil over five times the upper limit of normal levels (5 mg/dL), INR ≥1.5, or prothrombin time activity <40% (11). Patients were excluded if they were co-infected with other viruses (e.g., HAV, HCV, HDV, and HIV), consumed alcohol, had drug-induced liver diseases, were under treatment with antivirals or immunomodulating agents, or exhibited bleeding/infection. The study protocol was approved by the Ethics Committee of Huashan Hospital affiliated with Fudan University and the First Hospital of Quanzhou affiliated with Fujian Medical University.

### Animal model

Two C57BL/6 mice (8 weeks old, weighing 20 ± 1 g) were purchased from B&K Universal Group Ltd. (Shanghai, China). Animals were bred in a specific pathogen-free barrier facility. All experiments were approved by the Ethics Committee of the Shanghai Public Health Clinical Center and Institutes of Biomedical Science, Shanghai Medical College, Fudan University.

For the ACLF model, ConA (8 mg/kg, Sigma-Aldrich, USA) in 0.9% saline was injected into the retrobulbar angular vein five times every 2 days. Peripheral blood and liver tissues were sampled on the same days as the injections. Mice were euthanized after the last injection.

### Flow cytometry

For surface marker staining, peripheral blood mononuclear cells (PBMCs) and liver-infiltrating lymphocytes from HC, CHB, and HBV-ACLF patients were labeled with the following antibodies: anti-human BV510 CD33 (BioLegend), FITC CD11b (eBscience), APC HLA-DR (eBscience), PE/Cy7 CD14 (eBscience), BV421 CD15(eBscience), Percp-Cy5.5 BTLA (Biolegend), and LIGHT PE (BD Biosciences). For intracellular staining, cells were fixed and permeabilized using a Cytofix/Cytoperm Plus kit (BD Biosciences), then stained with PerCP-eFluor 710 anti-human indoleamine 2, 3-dioxygenase (IDO) (eBioscience) and PE IL-10 (eBioscience).

Liver, spleen, bone marrow-infiltrating lymphocytes, and whole blood from ACLF mice and normal mice were labeled with the following antibodies: anti-mouse PE GR-1 (BioLengend), FITC CD11b (BioLengend), APC Ly-6c (BioLengend), and PE/Cy7 Ly-6G (BioLengend). After incubation for 30 min, cells were subjected to red blood cell lysis (BioLengend), washed twice with PBS, and gMDSC (CD11b^+^Ly-6C^-^Ly-6G^+^) frequency was analyzed.

### Immunofluorescence double-staining

Tissue samples were deparaffinized with xylene and ethyl alcohol, quickly rinsed with TBST buffer for 5 min, and blocked with 5% normal serum TBST for 1 h. Liver sections from HBV-ACLF patients were incubated with mouse monoclonal anti-CD15 (BioLengend, USA) and rabbit monoclonal anti-CD11b (Abcam, USA) antibodies at 4°C overnight. Liver and spleen sections from ACLF mice were incubated with rabbit monoclonal anti-Ly-6G (Servicebio, China), rabbit anti-CD11b (Servicebio, China), or rabbit anti-IDO (Biosynthesis Biotechnology Co., LTD, Beijing, China). Subsequently, all sections were incubated with their corresponding Alexa 488 and Alexa 555 antibodies (Jackson ImmunoResearch, West Grove, PA, USA), and then incubated again with DAPI (Sigma, CA, USA). The results were analyzed using an inverted Eclipse Ti-S microscope (Nikon, Japan).

### Induction of gMDSC expansion *in vitro*

PBMCs from healthy donors were cultured in a complete medium at 1 × 10^6^ cells/mL for 1, 3, 5, and 7 days with either HBV-ACLF and HC plasma, or various concentrations (5–50 ng/mL) of recombinant human (rh) tumor necrosis factor (rhTNF-α), interleukin-6 (rhIL-6), rhIL-1β, rhIL-22, rhIL-37, rhIL-10, and rhFGF2. All antibodies were purchased from PeproTech, and rhIL-37 was purchased from R&D Systems. The medium and cytokines were refreshed every other day. Flow cytometry was used to determine the number of gMDSC.

Next, PBMCs from healthy donors were cultured in complete medium and split into the following groups: (a) control (no treatment); (b) stimulant alone (50 ng/mL): rhIL-6 or rhTNF-α alone; (c) inhibitor alone: anti-TNF-α (10 μg/mL, eBscience), stat3 inhibitor (SH-4-54, 50 μmol/L, Selleck), or NF-kb inhibitor (QNZ, 20 μmol/L, Selleck); and (d) stimulant plus inhibitors. Each treatment lasted for 3 days. All experiments were performed six times.

### Chromatin immunoprecipitation

The analysis was performed using the ChIP Assay Kit (Upstate, Lake Placid, NY, USA). Briefly, 293t cells were sonicated until DNA fragments averaged between 200 and 1000 bp. Chromatin was subsequently immunoprecipitated with antibodies (2 μg) against IDO, and an equal amount of IgG was used as a negative control for nonspecific immunoprecipitation. Next, ChIP DNA fragments were purified, reverse-transcribed, and used as templates for PCR. The PCR products were analyzed using agarose gel electrophoresis. Antibodies used were anti-IDO antibody (Abcam) and normal rabbit IgG (Santa Cruz). Primers specific to the IDO region were as follows: F, TCTCGGGCTCAAGCAATTC; R, TTCCGTTTATCCAGTCATCTC.

### T cell proliferation and cytokine secretion assays

CD14^−^CD15^+^ cells were purified from PBMCs or TNF-α-treated PBMCs with CD14-negative and CD15-positive selection kits (Miltenyi Biotec). Additionally, CFSE-labeled (Invitrogen) Pan T cells were purified from HC using magnetic beads (Miltenyi Biotec). The two sets of purified cells were co-cultured in various ratios and stimulated with human T-activator CD3/CD28 (eBscience) and/or IDO inhibitor (INCB024360, Selleck) for 3 days. Cells were stained with PE-Cy7 anti-human CD8 (eBscience), PE anti-human CD4 (eBscience), and FITC anti-human CD3 (eBscience). T cell proliferation was analyzed using flow cytometry.

For intracellular cytokine detection, co-cultured cells were stimulated with 50 ng/mL PMA (Sigma-Aldrich), 1 μg/mL ionomycin (Sigma-Aldrich), and 5 μg/mL BFA (Sigma-Aldrich) for 5 h. T cells were permeabilized and stained with APC anti-human IFN-γ (eBscience), PE anti-human IL-2 (eBscience), and PE-Cy7 anti-human TNF-α (eBscience).

### Whole-transcriptome library preparation and sequencing

Total RNA of gMDSC (CD14^−^CD15^+^) from HC and HBV-ACLF patients was extracted using the miRNeasy Mini Kit (Qiagen), then purified using the RNA Clean XP Kit (Beckman Coulter, Inc. CA, USA). Strand-specific sequencing libraries were generated using Superscript II Reverse Transcriptase (Invitrogen, USA). An Illumina HiSeq 2000 platform was used for RNA-seq.

### Whole blood phagocytosis assay

The phagocytosis assay was performed using the pHrodo *Escherichia coli* Green BioParticle Phagocytosis Kit (Invitrogen, Paisley, UK). Whole blood (100 μL) was added to 20 μL pHrodo *E. coli* and incubated for 15 min at 37°C under dark conditions. Next, 1 μL each of anti-human PE CD33 (eBscience), FITC CD11b (eBscience), APC HLA-DR (eBscience), PE/Cy7 CD14 (eBscience), and BV421 CD15 (eBscience) were added. The mixture was incubated again for 30 min at 4°C before 2 mL erythrocyte lysate (Invitrogen, Paisley, UK) was added. After a third incubation for 8 min at room temperature, cells were washed twice with PBS. Cells were acquired using a flow cytometer (CytoFLEX S, Beckman, USA).

### Statistical analyses

Data are expressed as means ± SEM. Significant differences were tested using the Mann–Whitney U, Wilcoxon, or Kruskal-Wallis tests, or one-way ANOVA, as appropriate. Correlation analysis was performed using Spearman’s correlation coefficients. Graphs were drawn in GraphPad Prism 8.0 (San Diego, California, USA).

## Results

### Patient characteristics

Patients with HBV-ACLF were older than those with chronic hepatitis B (CHB) and HC (for clinical characteristics, see **Table 1**). Moreover, patients with HBV-ACLF showed significantly higher physiological and biochemical indicators of liver injury (total bilirubin, TBil; alanine aminotransferase, ALT; aspartate aminotransferase, AST; alkaline phosphatase, ALP; γ-Glutamyl transferase, GGT; prothrombin time, PT; and international normalized ratio, INR) than patients with CHB, but markedly lower compensatory indices of liver function (ALB, Hgb, and PLT) and virological parameters (HBV DNA). In addition, 60.38% of patients with HBV-ACLF developed bacterial infections during their hospital stay, and 49.09% of patients with HBV-ACLF showed a poor prognosis.

**Table 1:** Demographic and clinical characteristics of subjects.

Group	NC (*n* = 40)	CHB (*n* = 84)	HBV-ACLF (*n* = 53)
Sex (Male, %)	28 (70.00%)	61 (72.62%)	43 (81.13%)
Age (years)	35.14 ± 3.59	35.79 ± 1.11	45.59 ± 1.65 *^,‡^
HBV DNA (Lg IU/ml)	–	5.16 ± 0.28	3.74 ± 0.21 *
Albumin (g/L)	–	45.31 ± 0.52	36.29 ± 0.71 *
Total bilirubin (μmol/L)	–	15.14 ± 1.50	332.77 ± 30.04 *
Alanine aminotransferase (IU/L)	–	95.88 ± 11.31	297.44 ± 76.32 *
Aspartate aminotransferase (IU/L)	–	50.22 ± 5.49	228.39 ± 87.32 *
Alkaline phosphatase (U/L)	–	82.91 ± 4.13	130.84 ± 4.96 *
γ-Glutamyl transferase (U/L)	–	39.84 ± 6.60	85.65 ± 14.93 *
Creatinine (μmol/L)	–	65.09 ± 2.71	83.87 ± 11.95
White blood cell count (10^9^/L)	–	5.86 ± 0.24	5.61 ± 0.36
Neutrophil count (10^9^/L)	–	3.51 ± 0.24	3.28 ± 0.24
Hemoglobin (g/L)	–	143.96 ± 3.61	109.31 ± 3.58 *
Platelet count (10^9^/L)	–	191.29 ± 8.08	80.24 ± 7.16 *
Prothrombin time (s)	–	11.48 ± 0.16	24.04 ± 1.30 *
International normalized ratio	–	1.02 ± 0.01	2.08 ± 0.09 *
C-reactive protein (mg/L)	–	–	15.67 ± 2.37
Procalcitonin (ng/ml)	–	–	0.78 ± 0.14
Child-pugh score	–	–	10.35 ± 0.22
MELD score	–	–	22.46 ± 1.21
Good prognosis n (%)	–	–	27 (50.94%)
Bacterial infection n (%)	–	–	32 (60.38%)
Ascites n (%)	–	–	33 (62.26%)
Hepatic encephalopathy n (%)	–	–	4 (7.55%)
Hyperplenism n (%)	–	–	40 (75.47%)

Results are expressed as medians and interquartile ranges. * Significant difference comparing HBV-ACLF to CHB patients and ^‡^ comparing HBV-ACLF to NC.

### Increased proportion of gMDSC in patients with HBV-ACLF

**Figure 1:**
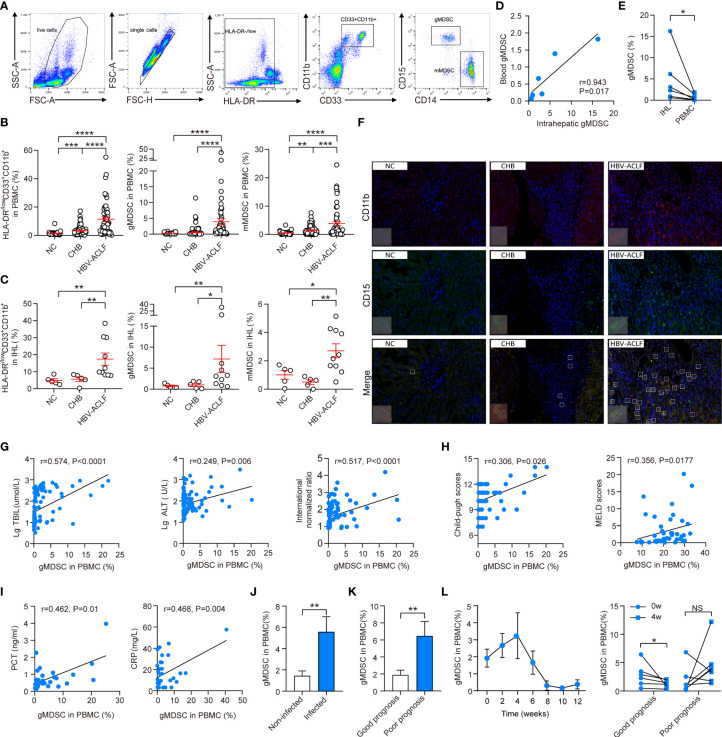
The proportion of gMDSC was correlated with disease progression, prognosis, and infectious complications in HBV-ACLF patients. **(A)** Sequential gating strategy for gMDSC (HLA-DR^−/low^CD33^+^CD11b^+^CD14^−^CD15^+^) and mMDSC (HLA-DR^−/low^CD33^+^CD11b^+^CD14^+^) identification using flow cytometry from freshly isolated PBMCs. The proportion of gMDSC was calculated as a percentage of live PBMCs. **(B, C)** Cumulative dot plots of myeloid cells (HLA-DR^−/low^CD33^+^CD11b^+^); gMDSC and mMDSC in peripheral blood **(B)** and intrahepatic lymphocytes **(C)**. **(D, E)** Cumulative data depicting the relationship **(D)** and differences **(E)** between circulating and intrahepatic gMDSC. **(F)** Representative double epitope immunostaining of liver sections from HC, CHB, and HBV-ACLF participants for CD11b (red) and CD15 (green). Scale bar, 70×. **(G-I)** Correlation of circulating gMDSC proportion with biochemical indices of liver injury **(G)**, prognosis **(H)**, and inflammation **(I)**. **(J, K)** Histogram of gMDSC as a percentage of PBMCs, categorized by infection **(J)** and prognosis **(K)** in HBV-ACLF patients. **(L)** Changes in gMDSC during disease progression of HBV-ACLF patients. Error bars, mean ± SEM; one-way ANOVA followed by Fisher’s LSD multiple-comparison test **(B, C)**; Mann–Whitney U test **(J, K)**; Wilcoxon test **(E, L)**; and Spearman tests **(G-I)**; *P <.05, **P <.01, ***P <.001, and ****P <.0001. NS, no significance.

We classified MDSCs as HLA-DR^−/low^CD33^+^CD11b^+^CD14^−^CD15^+^ (gMDSC) and HLA-DR^−/low^CD33^+^CD11b^+^CD14^+^ cells (mMDSC, **Figure 1A**) (6). Myeloid cells (HLA-DR^−/low^CD33^+^CD11b^+^) markedly expanded in HBV-ACLF (11.45 ± 1.64%) compared with CHB (3.26 ± 0.33%, P < 0.001) and HC (1.32 ± 0.20%, P < 0.001). The proportion of myeloid cells was also higher in patients with CHB than in HC (P < 0.001). Similarly, gMDSC and mMDSC proportions in PBMCs of patients with HBV-ACLF (gMDSC: 3.94 ± 0.93%, mMDSC: 3.87 ± 0.78%) were also significantly higher than in PBMCs of patients with CHB (0.60 ± 0.17%, P < 0.001; 1.49 ± 0.17%, P = 0.006) and HC (0.24 ± 0.03%, P < 0.001; 0.51 ± 0.09%, P < 0.001). In addition, patients with CHB had a higher frequency of mMDSC, but not of gMDSC, than HC (P = 0.001, **Figures 1B**, **S1A**), suggesting that gMDSC are only specifically increased in the peripheral blood of patients with HBV-ACLF and may be an HBV-ACLF predictor.

Unexpectedly, we found that the proportion of HLA-DR^−/low^CD33^+^CD11b^+^ myeloid cells, along with gMDSC and mMDSC, were significantly higher in intrahepatic lymphocytes (IHL) of patients with HBV-ACLF (myeloid cells: 17.38 ± 3.63%; gMDSC: 7.20 ± 3.25%; mMDSC: 2.71 ± 0.49%) than in IHL of patients with CHB (5.59 ± 1.41%, P = 0.005; 1.02 ± 0.36%, P = 0.04;0.50 ± 0.16%, P = 0.005) and HC (4.82 ± 1.05%, P = 0.005; 0.75 ± 0.19%, P = 0.008; 1.01 ± 0.31%, P = 0.04). However, the difference between HC and patients with CHB was not significant (p > 0.05, **Figures 1C**, **S1B**). Additionally, interhepatic gMDSC were present in significantly higher proportions than peripheral blood gMDSC, and the two were positively correlated (r = 0.943, P < 0.017, n = 5, **Figures 1D, E**). Immunofluorescence confirmed that the number of CD11b^+^CD15^+^ double-positive cells was higher in the liver of patients with HBV-ACLF than that in the liver of patients with CHB and HC (**Figure 1F**). These results suggest that gMDSC were expanded in the circulation and liver of patients with HBV-ACLF.

### The proportion of gMDSC was correlated with disease progression, prognosis, and infectious complications in patients with HBV-ACLF

After assessing the relationship with clinical parameters, we found that the gMDSC proportion was positively correlated with liver injury (TBil, ALT, and INR, **Figure 1G**), prognosis (Child-Pugh and MELD scores, **Figure 1H**), and inflammation (PCT and CRP, **Figure 1I**).

Next, we tested the association between gMDSC and infectious complications. We found that the gMDSC proportion was higher in co-infected patients than in non-infected patients (**Figure 1J**). Furthermore, the poor prognosis group had more gMDSC than the good prognosis group (**Figure 1K**). Details of the two groups are described in one of our previous studies (12). After comprehensive treatment, gMDSC proportions in patients with HBV-ACLF gradually increased over 4 weeks, then fell rapidly and remained low for a long time. Importantly, this decrease was driven by patients with HBV-ACLF with a good prognosis, as we did not observe a significant change in patients with HBV-ACLF with a poor prognosis at 4 weeks (**Figure 1L**). These findings further suggest that gMDSC are likely to reflect impaired immunity against bacterial infection and may be a new predictor for disease prognosis or co-infection.

### Granulocytic MDSCs exhibit immunoenhanced phenotype

**Figure 2:**
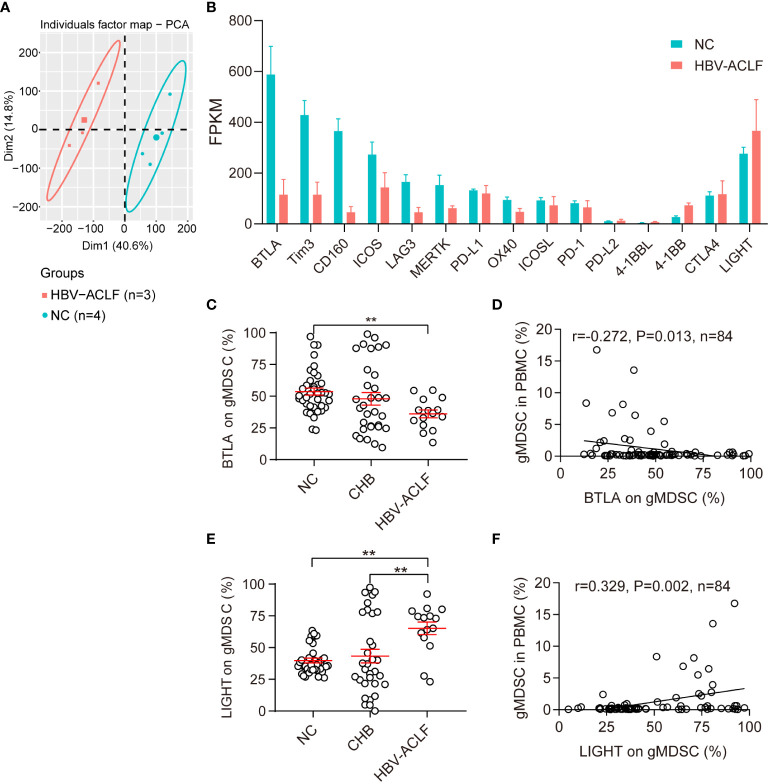
Phenotypic characteristics of gMDSC. **(A, B)** Differentially expressed genes in gMDSC from HC and HBV-ACLF patients. **(C, E)** Cumulative data for expression of BTLA (co-inhibitory molecule, **C**) and LIGHT (co-stimulatory molecule, **E**) in circulating gMDSC from patients with chronic HBV infection. **(D, F)** Correlation of circulating gMDSC proportion with BTLA **(D)** and LIGHT **(F)** expression in gMDSC. Error bars, mean ± SEM; one-way ANOVA followed by Fisher’s LSD multiple-comparison test **(B, D)** and Spearman tests **(C, E)**; *P <.05, **P <.01.

To characterize the functional phenotype of gMDSC, we examined their immune checkpoint expression profiles using RNA sequencing (RNA-seq). Compared with HC, patients with HBV-ACLF had lower expression of co-inhibitory molecules (BTLA, Tim3, CD160, LAG3, MERTK, PD-1, PD-L1, PD-L2, OX40) and two co-stimulatory molecules (ICOS, ICOSL), but higher expression of two other co-stimulatory molecules (4-BB and LIGHT, **Figures 2A, B**).

Next, we used flow cytometry to test BTLA and LIGHT expression for further verification of gMDSC phenotypic characteristics. The results showed that gMDSC in patients with HBV-ACLF had a significantly lower BTLA expression than HC cells, but a significantly higher LIGHT expression than gMDSC in HC and patients with CHB (**Figures 2C, E**, **S1C, D**). Additionally, gMDSC proportions among PBMCs were positively correlated with LIGHT expression (r = 0.329, P = 0.002, **Figure 2F**) and negatively correlated with BTLA expression (r = -0.272, P = 0.013, **Figure 2D**). LIGHT and BTLA are positive and negative immune checkpoint molecules, respectively. gMDSC of HBV-ACLF patients showed high expression of LIGHT molecules and low expression of BTLA molecules, suggesting that the gMDSC phenotype is immunoenhanced, rather than immunoparalytic.

### Expansion of gMDSC was dependent on TNF-α

**Figure 3:**
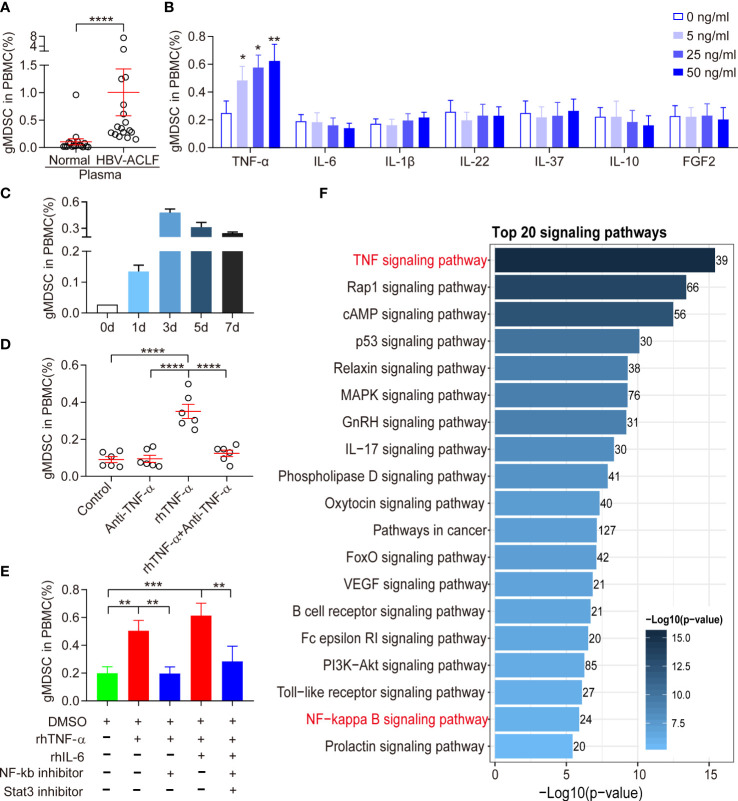
Expansion of gMDSC was dependent on TNF-α. **(A)** Cumulative dot plots of gMDSC proportion when PBMCs were exposed to plasma from HC or HBV-ACLF patients. **(B)** Histogram of gMDSC proportion when PBMCs were exposed to different concentrations of recombinant human (rh) cytokines (TNF-α, IL-6, IL-1β, IL-22, IL-37, IL-10, and FGF2). **(C)** Histogram of gMDSC proportion after rhTNF-α stimulated HC PBMCs for 1, 3, 5, and 7 days (n = 3). **(D, E)** Cumulative dot plots of gMDSC proportion when HC PBMCs were co-cultured with rhTNF-α, rhIL-6, anti-TNF-α, NF-kb inhibitor, and Stat3 inhibitor for 3 days. **(F)** TNF signaling was the most active pathway in gMDSC purified from HBV-ACLF. Error bars, mean ± SEM; Wilcoxon test **(A–E)**; *P <.05, **P <.01, ***P <.001, and ****P <.0001.

Because the inflammatory microenvironment may support MDSC expansion (13), we investigated the effect of HBV-ACLF plasma on gMDSC. Exposure of PBMCs to HBV-ACLF plasma resulted in a higher gMDSC to PBMC ratio than exposure to HC plasma (**Figure 3A**).

Patients with HBV-ACLF had higher levels of pro-inflammatory cytokines (TNF-α, IL-6, IL-1β, IL-22, and FGF2) and anti-inflammatory cytokines (IL-10 and IL-37) than HC or patients with CHB (unpublished data). Hence, we also investigated the role of cytokines in gMDSC expansion. We used recombinant human (rh) IL-1β, rhIL-6, rhIL-22, rhIL-10, rhIL-37, rhFGF2, and rhTNF-α to stimulate HC PBMC *in vitro*. Only rhTNF-α significantly increased gMDSC expansion in a dose-dependent manner, while rhIL-1β, rhIL-22, rhIL-6, and rhIL-37 had no effect (**Figure 3B**). We observed that the gMDSC proportion was higher on day 3 than on days 5 or 7 (**Figure 3C**). Additionally, anti-TNF-α blocked rhTNF-α-induced gMDSC expansion (**Figure 3D**). Interestingly, the combination of rhIL-6 and rhTNF-α enhanced gMDSC expansion, an effect blocked by Stat3 and NF-kb inhibitors (**Figure 3E**). Notably, TNF signaling was the most active pathway in gMDSC purified from HBV-ACLF patients (**Figure 3F**). These results suggest that circulating cytokines, particularly TNF-α, induce gMDSC expansion.

### Granulocytic MDSCs inhibit cytokine secretion and T cell proliferation *via* IDO/IL-10 pathway

**Figure 4:**
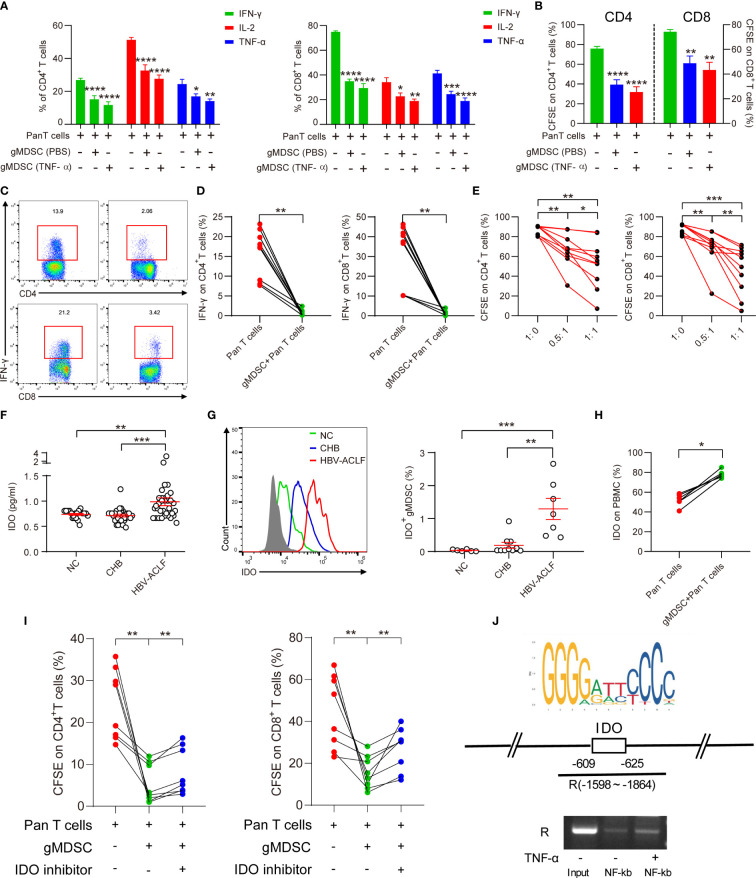
gMDSC inhibit cytokine secretion and T cell proliferation *via* IDO/IL-10 signaling pathway. **(A, B)** Percentage of cytokines (IFN-γ, IL-2, and TNF-α; A) and proliferation of CD4^+^ and CD8^+^ T cells **(B)** were detected in Pan T cells co-cultured with gMDSC from PBMCs, plus or minus TNF-α stimulation. **(C, D)** Representative FACS **(C)** and cumulative dot plot **(D)** of IFN-γ-producing CD4^+^ and CD8^+^ T cell frequency after HC Pan T cells were co-cultured with or without gMDSC isolated from HBV-ACLF patients. **(E)** Cumulative dot plot of CD4^+^ and CD8^+^ T cell proliferation after Pan T cells were co-cultured with gMDSC from HBV-ACLF patients at different ratios. **(F)** Soluble IDO was detected and compared across groups using ELISA. **(G)** Representative FACS and cumulative dot plot of IDO^+^ gMDSC. **(H)** Cumulative plot of IDO as a percentage of PBMCs in Pan T cells co-cultured with or without gMDSC from HBV-ACLF patients. **(I)** Cumulative data for CD4^+^ and CD8^+^ T cell proliferation in co-cultured gMDSC with or without exposure to IDO inhibitor. **(J)** ChIP-qPCR assay confirmed that NF-kb, a downstream target of TNF-α, binds to the IDO promoter region. *P <.05, **P <.01, ***P <.001, and ****P <.0001.

After incubating HC PBMCs with or without TNF-α for 3 days, we separated gMDSC using magnetic beads and co-cultured them with autologous Pan T cells. The isolated gMDSC (with or without TNF-α) significantly decreased intracellular cytokine production (IFN-γ, IL-2, and TNF-α), and decreased CD4^+^ and CD8^+^ T cell proliferation. TNF-α-induced gMDSC had a slightly stronger inhibitory effect than non-TNF-α-induced gMDSC (**Figures 4A, B**). Next, to assess whether gMDSC impair T cell functions under pathological conditions, we co-cultured HC Pan T cells with or without gMDSC from patients with HBV-ACLF, and then analyzed cytokine secretion and proliferation of Pan T cells. The results showed that gMDSC from patients with HBV-ACLF significantly inhibited intracellular IFN-γ production in CD4^+^/8^+^ T cells (**Figures 4C, D**) and markedly decreased T cell proliferation in a dose-dependent manner (**Figure 4E**).

Previous studies have shown that several factors, including Arg1 and IL-10, are responsible for gMDSC suppression of T cells (6, 14). Our previous research has suggested that mMDSC impair T cell function through the IDO pathway (15). Therefore, we aimed to identify whether the IDO and IL-10 pathways are responsible for gMDSC suppression of T cell function. The results of ELISA showed that patients with HBV-ACLF had higher soluble IDO (sIDO) and IL-10 levels than HC and patients with CHB (**Figures 4F**, **S2A, B**). Additionally, sIDO levels were positively correlated with gMDSC proportion, Child-Pugh scores, MELD scores, INR, and TBil levels, but negatively correlated with PLT levels (**Figures S3A, B, C**). These patterns are consistent with gMDSC expression trends and correlations.

Intracellular staining then confirmed that IDO and IL-10 expression in gMDSC was significantly higher in patients with HBV-ACLF than in HC and patients with CHB (**Figures 4G**, **S2C**). In addition, Pan T cells co-cultured with gMDSC from patients with HBV-ACLF had higher IDO and IL-10 expression than Pan T cells alone (**Figures 4H**, **S2A, D**). Furthermore, IDO inhibitor 1 efficiently restored CD4^+^ and CD8^+^ T cell proliferation in a co-culture of gMDSC with autologous T cells (**Figure 4I**).

We analyzed JASPAR datasets and conducted a ChIP assay to investigate whether NF-kb, a downstream target of TNF-α, regulates IDO expression through binding to its promoter region. The results of ChIP-qPCR confirmed that NF-kb binds to a site in the IDO promoter region located at approximately -609 to -625 (**Figure 4J**). Thus, gMDSC seem to impair T cell function in an IDO-dependent manner.

### Increased gMDSC and IDO^+^gMDSC proportions in ACLF mouse model

**Figure 5:**
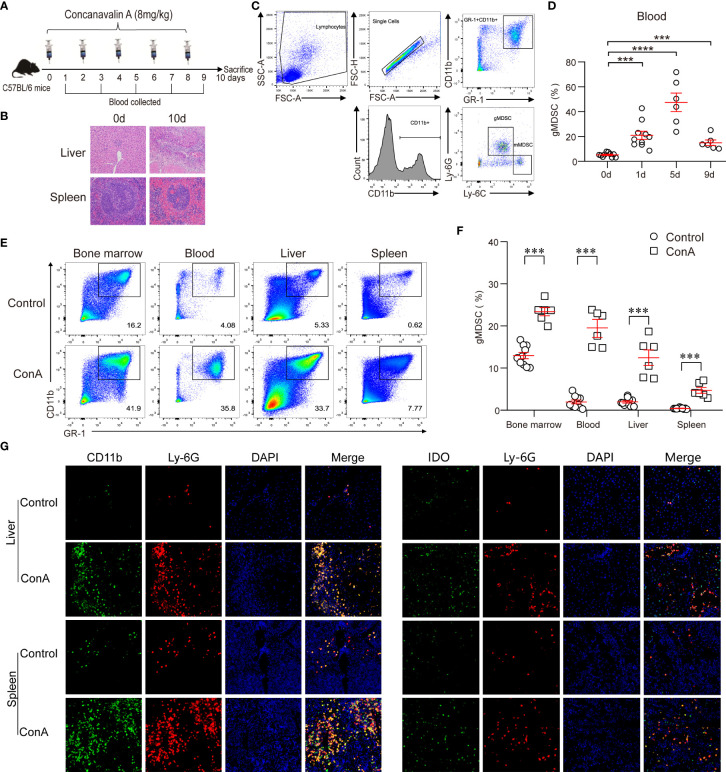
Increased gMDSC and IDO^+^gMDSC proportions in the ACLF mouse model. **(A, B)** Flow chart **(A)**, along with liver and spleen pathology **(B)** of the ACLF mouse model generated from continuous ConA injection. **(C)** Representative FACS plot of gMDSC and mMDSC. **(D)** Dynamic changes to gMDSC after continuous ConA injection. **(E, F)** Representative FACS **(E)** and cumulative dot plot **(F)** of gMDSC in bone marrow, blood, liver, and spleen of ACLF mice. **(G, H)** Representative double epitope immunostaining of liver and spleen tissue from ACLF mice for CD11b (green) and Ly-6G (red); Ly-6G and IDO; Scale bar, 70×. ***P <.001, and ****P <.0001.

Previous studies have shown that an ACLF animal model can be successfully established using continuously repeated concanavalin A (ConA) stimulation (16). Using the same method, we successfully established an ACLF mouse model with continuous low-dose ConA (8 mg/kg) exposure (**Figure 5A**). On day 10, H&E staining of the liver and spleen showed sub-massive or massive tissue necrosis, accompanied by many inflammatory cell immersions, suggesting that the ACLF model was successfully established (**Figure 5B**). We divided murine MDSC into CD11b^+^Ly-6G^−^Ly-6C^+^ mMDSC, and CD11b^+^Ly-6G^+^Ly-6C^-^ gMDSC (**Figure 5C**) (17). In the ACLF model, gMDSC increased gradually, peaked on day 5, and then gradually decreased, but were still higher than those in the control group (**Figure 5D**). On day 4, ACLF mice had a significantly higher gMDSC proportion in bone marrow, peripheral blood, liver, and spleen than the control group. We also found that bone marrow had the highest proportion of gMDSC, followed by peripheral blood, liver, and spleen (**Figures 5E, F**). Additionally, immunofluorescence showed that the number of double-positive CD11b^+^Ly-6G^+^ cells was higher in the liver and spleen of the ACLF group than that of the control group (**Figure 5G**). Another immunofluorescence assay then indicated that the number of double-positive Ly-6G ^+^IDO^+^ cells was higher in the liver and spleen of ACLF mice than that of control mice (**Figure 5H**), indicating increased IDO expression in ACLF gMDSC.

### Granulocytic MDSCs from patients with HBV-ACLF display impaired phagocytosis of *E. coli*

**Figure 6:**
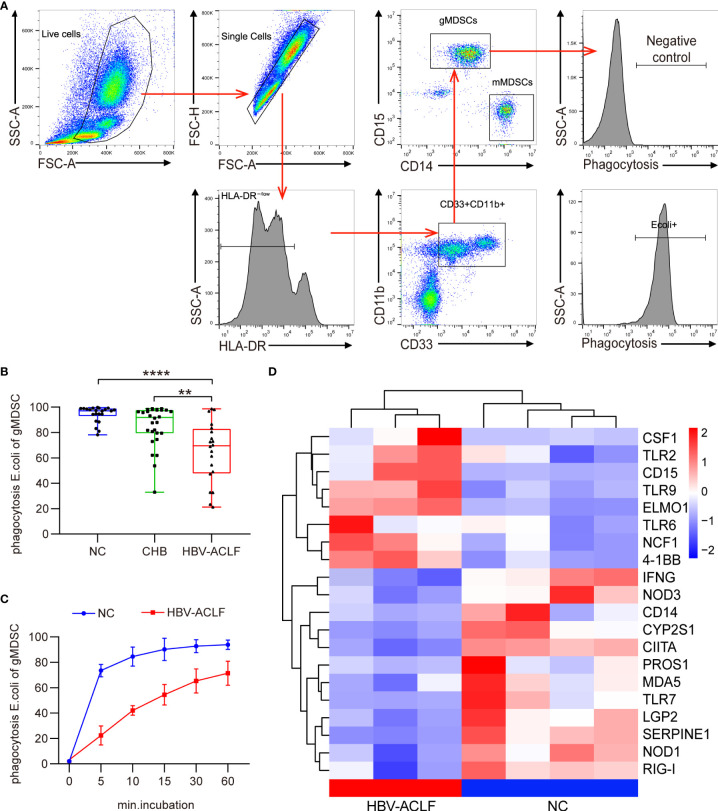
gMDSC from HBV-ACLF patients display impaired phagocytosis of *E. coli*. **(A)** Representative FACS **(A)** and cumulative dot plot **(B)** showing gMDSC phagocytosis of *E. coli* in HC, CHB, and HBV-ACLF groups. **(C)** Time-dependent analysis of phagocytosis assay in HC and HBV-ACLF patients. **(D)** Heat map of differential mRNA expression in gMDSC from HC (n = 4) and HBV-ACLF patients (n = 3).

Previous studies have shown that mMDSC of patients with ACLF exhibit a marked and persistent deficiency in bacterial uptake and clearance (5). We thus performed a phagocytosis assay using *E. coli* to test whether gMDSC from patients with HBV-ACLF displayed the same problem. Patients with HBV-ACLF had significantly fewer gMDSC with phagocytic ability than HC and patients with CHB (**Figures 6A, B**). Furthermore, the capacity of gMDSC in patients with HBV-ACLF had a consistently lower capacity to phagocytose *E. coli* than HC gMDSC (**Figure 6C**).

To explore the mechanism behind impaired phagocytosis in patients with HBV-ACLF, we analyzed gMDSC gene expression profiles across HC and patients with HBV-ACLF using RNA-seq. Innate immune cells recognize pathogen-associated molecular patterns through expressing pattern recognition receptors (PRRs), including toll-like receptors (TLR), retinoic-acid-inducible gene I-like receptors (RLR), and Nod-like receptors (NLR). Compared with control gMDSC, gMDSC from patients with HBV-ACLF had a lower expression of RLR genes (*RIG-1*, *MDA5*, and *LGP2*), NLR genes (*NOD1*, *NOD3*, and *CIITA*), TLR genes (*TLR7*), and IFN-γ (**Figure 6D**). These findings indicate that multiple defective PRRs exist in HBV-ACLF gMDSC and are potential therapeutic targets.

## Discussion

Hospitalized patients with HBV-ACLF are prone to secondary bacterial or fungal infections that significantly increase mortality risk. In this study, we successfully clarified aspects of the molecular mechanisms underlying susceptibility to infection associated with HBV-ACLF. Specifically, we found that gMDSC proportions in the peripheral blood and liver of patients with HBV-ACLF increased significantly. Moreover, the increase was positively correlated with disease severity, prognosis, and infection complications. Through acting on the IDO/IL-10 pathway, gMDSC inhibited T cell proliferation and IFN-γ production, attenuating antimicrobial response. Furthermore, the ability of gMDSC to phagocytose bacteria was impaired in patients with HBV-ACLF. These two factors are likely the primary reasons behind the increased susceptibility to infection among this cohort.

In chronic hepatitis B, HBsAg or HBeAg maintain persistent HBV infection and inhibit T cell response through promoting monocyte differentiation into mMDSC (15, 18). Additionally, hepatic stellate cell-induced gMDSC constrain nutrient supply to proliferating T cells as a means of moderating liver damage (6). Similar to our results on gMDSC from both patients and an ACLF mouse model, previous research also found that mMDSC expanded in HBV-ACLF (or ACLF) patients and were closely associated with disease severity and progression (5, 19), but impairment of their phagocytic capacity increased infection risk (5). We demonstrated that immune-enhanced functional phenotypes of gMDSC resulted in their elevation, being associated with a higher risk of infection and poor prognosis. Our findings indicate that gMDSC may be useful as biomarkers of infection in patients with HBV-ACLF.

Although TNF-α signaling promotes MDSC migration and differentiation in general (20–22), it remains unknown whether TNF-α induces gMDSC in the inflammatory microenvironment of HBV-ACLF. Many previous studies and our unpublished data have confirmed that patients with HBV-ACLF display markedly increased TNF-α levels (23, 24). Here, we provide further evidence that TNF-α and other inflammatory cytokines promote gMDSC expansion. We also show that circulating and TNF-α-induced gMDSC display immunosuppressive functions.

Because of its involvement in inflammation and peripheral tolerance, IDO is commonly used as a therapeutic target for cancers and autoimmune diseases (25). Our previous study showed that mMDSC act on the IDO signaling pathway to suppress T cell responses and maintain persistent HBV infection (15). Here, we found that gMDSC IDO levels and IDO expression increased synchronously in the peripheral blood and liver of HBV-ACLF patients, as well as in the liver of ACLF mice. Elevated IDOs in all these cases were positively correlated with HBV-ACLF severity and prognosis. Additionally, exposure to an IDO inhibitor restored T cell proliferation in gMDSC isolated from HBV-ACLF patients, suggesting that gMDSC act through IDO to dampen the antimicrobial response. More importantly, this study is one of the first to empirically demonstrate that TNF-α induces IDO production in addition to promoting gMDSC expansion.

Another factor contributing to increased infection risk in patients with HBV-ACLF is decreased pathogen-clearing ability. Previous studies have shown that the phagocytic capacity of mMDSC decreases continuously or is lost in ACLF (5). Experiments with RNA-seq have revealed that gMDSC are a mature type of neutrophils, potent phagocytes similar to monocytes (26). Here, we confirmed that gMDSC phagocytic ability decreased persistently in HBV-ACLF patients, likely due to the presence of multiple defective PRRs.

This study had some limitations. First, no well-established method exists for inducing ACLF, especially in the ACLF mouse model with a background of HBV infection. Second, while we found that elevated gMDSC may indicate an increased risk of infection and death, the lack of accurate biomarkers or antibodies meant that we could not target gMDSC for removal. Thus, it was impossible to obtain direct evidence of whether gMDSC clearance reduces mortality and infection rates.

In conclusion, inflammatory cytokines, especially TNF-α, induced gMDSC expansion and promoted IDO production, thereby inhibiting the antimicrobial immune response in HBV-ACLF. Although the proportion of gMDSC rose in HBV-ACLF patients, their phagocytic capacity was impaired. These two deficiencies explain why patients with HBV-ACLF are susceptible to infection and provide possible targets for immunotherapy.

## Data availability statement

The data presented in the study are deposited in the CNSA repository (https://db.cngb.org/cnsa/), accession number CNP0003467.

## Ethics statement

The studies involving human participants were reviewed and approved by Ethics Committee of Huashan Hospital affiliated with Fudan University and the First Hospital of Quanzhou affiliated with Fujian Medical University. The patients/participants provided their written informed consent to participate in this study. The animal study was reviewed and approved by Ethics Committee of Huashan Hospital affiliated with Fudan University. Written informed consent was obtained from the owners for the participation of their animals in this study.

## Author contributions

Study concept and design: XY, JS, FY, RM, ZJS, JZ. Acquisition, analysis, and interpretation of data: XY, JS, FY, ZQS, LR, JZ. Material support: SY, QH, LZ, YY, XD, YH, HZ, ZLS, MZ. Drafting of the manuscript: XY, JS, FY. Critical revision of the manuscript for important intellectual content: XY, JS, FY, CQ, ZJS, JZ. Obtained funding: RM, XY, and JZ.

## Funding

This study was supported by the National Natural Science Foundation of China (81400625, 81871640, 82172255), the Natural Science Foundation of Fujian province (2019J01593, 2019Y9048), the Young and Middle-Aged Key Talent Training Project of Fujian Province (2020GGA076), the Shanghai Shen Kang Hospital Development Center (SHDC12019116), the Shanghai Municipal Science and Technology Major Project (ZD2021CY001), and the Shanghai Key Clinical Specialty Construction Program (ZK2019B24).

## Acknowledgments

We thank Jianqing Xu and Gan Zhao for excellent technical assistance and critical reading.

## Conflict of interest

The authors declare that the research was conducted in the absence of any commercial or financial relationships that could be construed as a potential conflict of interest.

## Publisher’s note

All claims expressed in this article are solely those of the authors and do not necessarily represent those of their affiliated organizations, or those of the publisher, the editors and the reviewers. Any product that may be evaluated in this article, or claim that may be made by its manufacturer, is not guaranteed or endorsed by the publisher.
