# Supplementary material for: Granulocytic myeloid-derived suppressor cells increase infection risk via the IDO/IL-10 pathway in patients with hepatitis B virus-related liver failure
Source: Front Immunol. 2023 Jan 4;13:966514. doi: 10.3389/fimmu.2022.966514 (PMC9847254; doi:10.3389/fimmu.2022.966514)
Supplement: Supplementary file 1 [file DataSheet_1.docx]

Supplementary Material

**Supplementary figure**


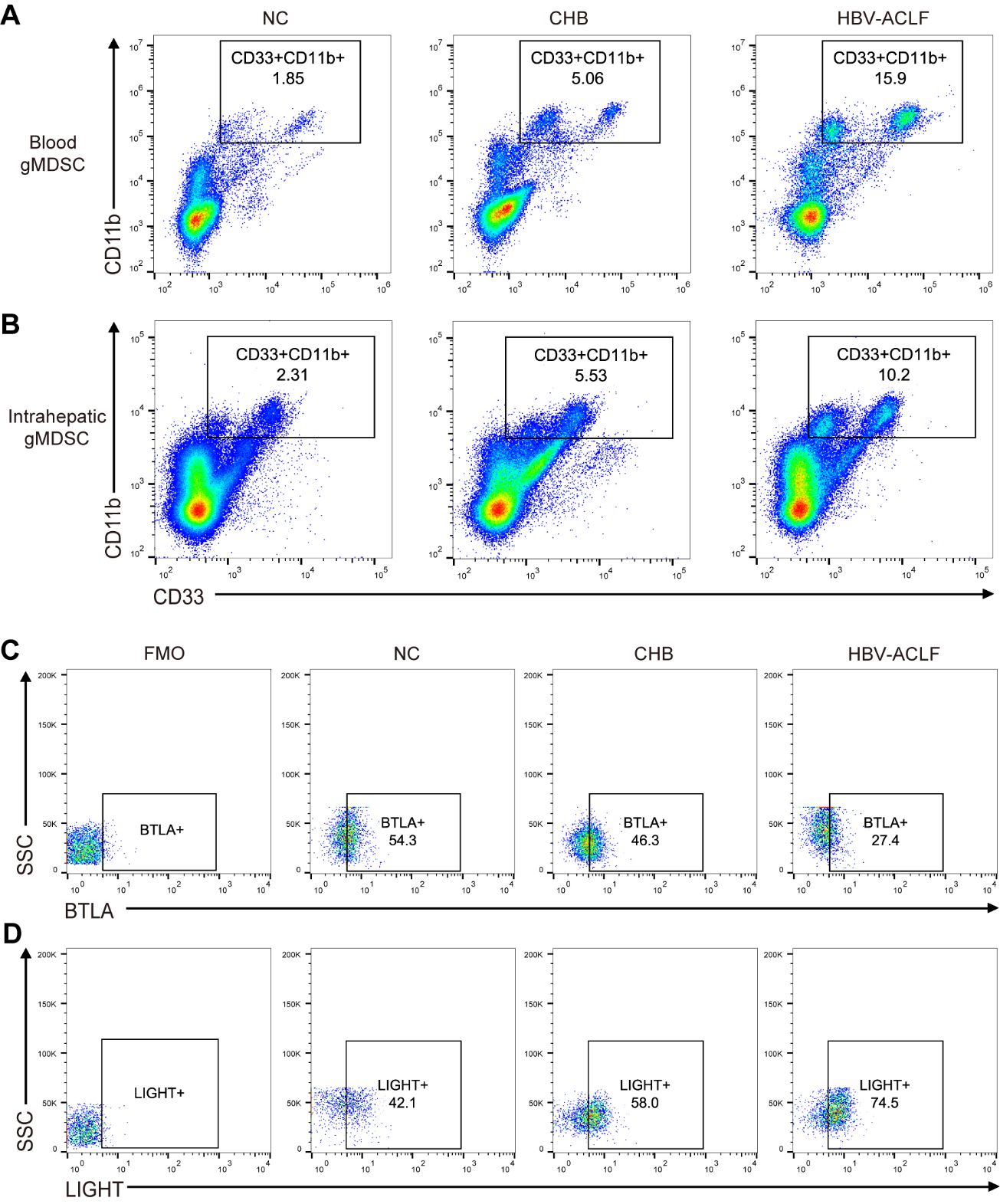


**Supplementary figure 1.** Representative FACS plot of gMDSC in the peripheral blood (A), and intrahepatic (B), as well as BTLA (C), and LIGHT (D) expression on gMDSC.


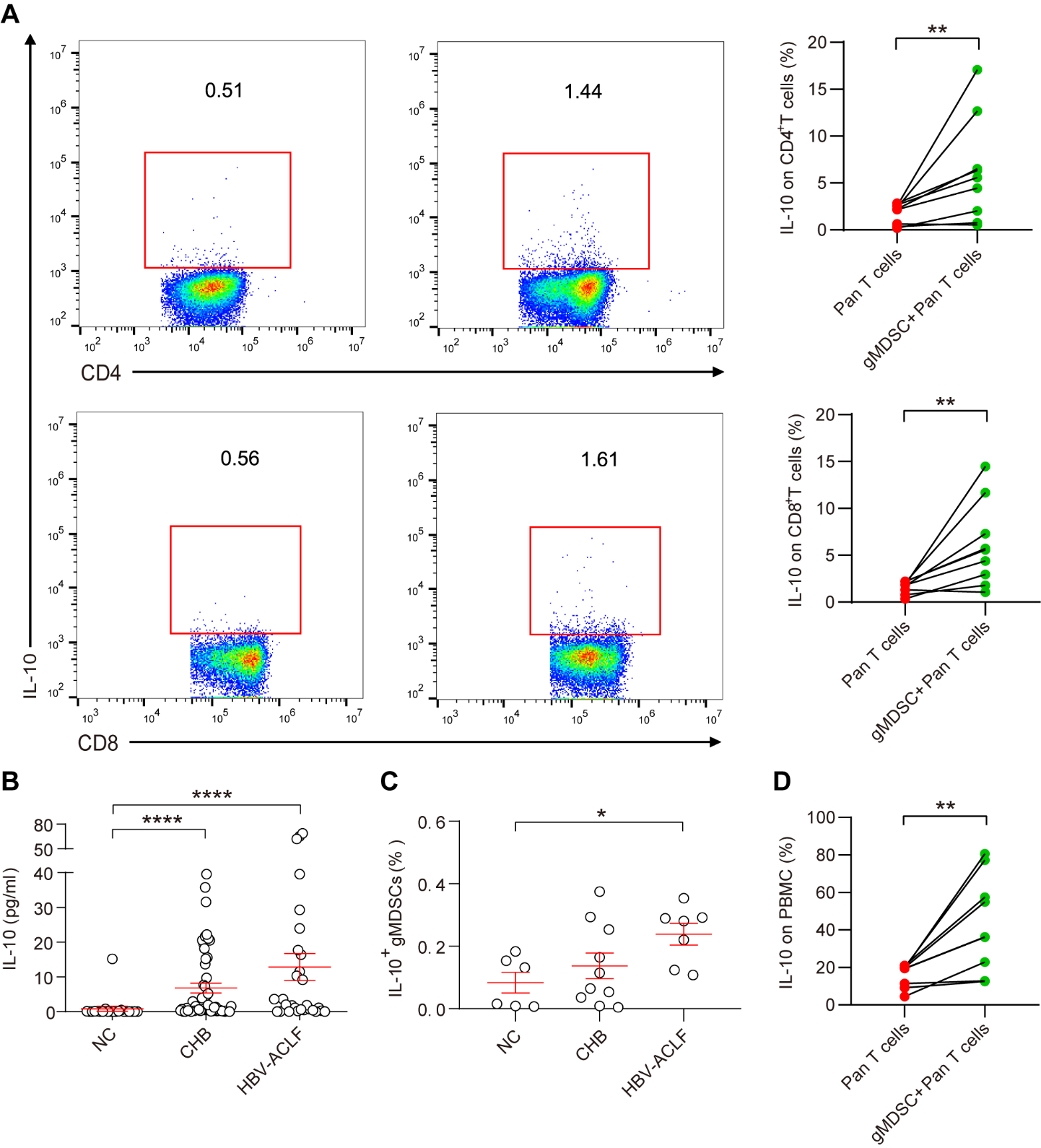
**Supplementary figure 2. gMDSC inhibit cytokine secretion and T cell proliferation via IL-10.** (A) Representative FACS and cumulative dot plot showing frequency of IL-10-producing CD4^+^ and CD8^+^ T when HC Pan T cells were co-cultured with or without gMDSC isolated from HBV-ACLF patients. (B) ELISA was used to detect IL-10 levels in different groups. (C) Representative FACS and cumulative dot plot of IL-10^+^ gMDSC. (D) Cumulative plot of IL-10 as a percentage of PBMCs in Pan T cells co-cultured with or without gMDSC from HBV-ACLF patients.


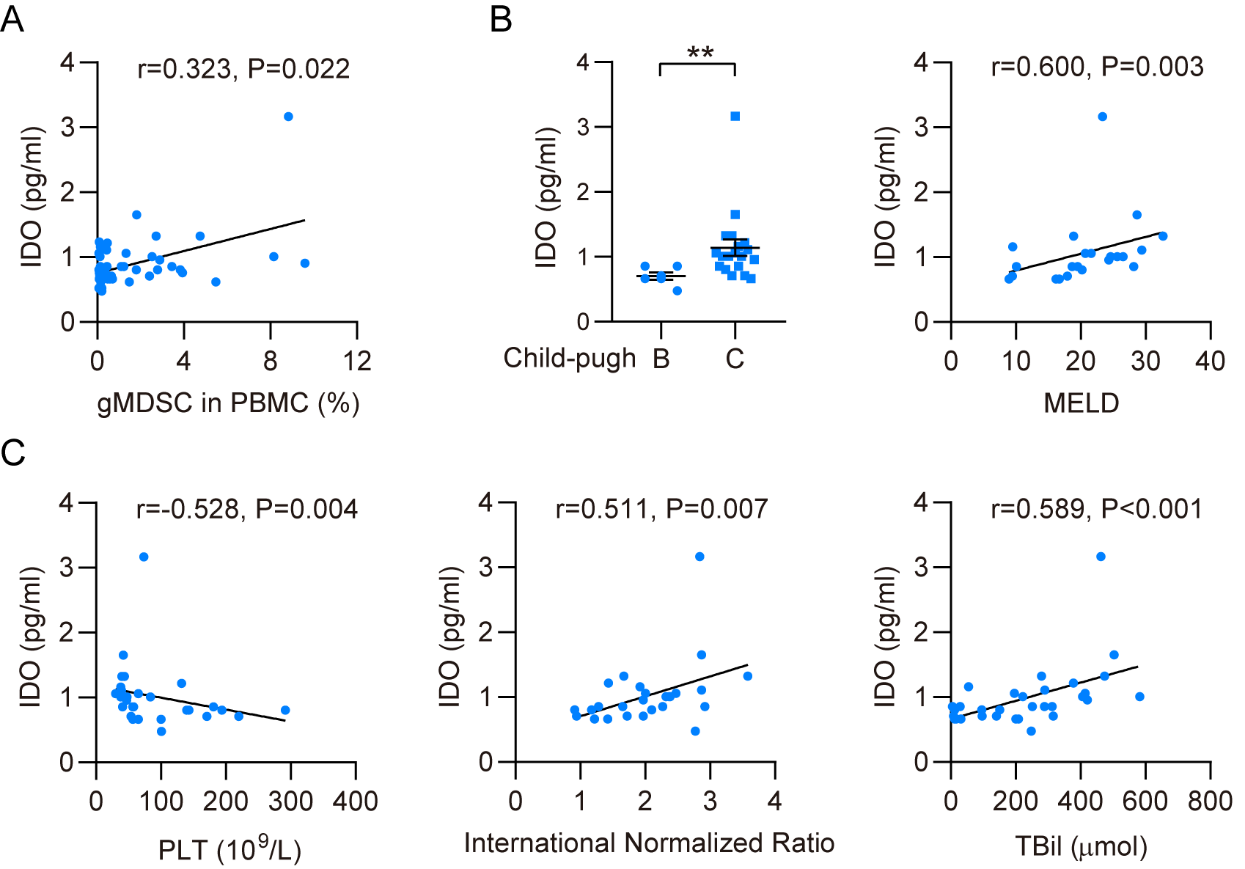


**Supplementary figure 2. IDO was associated with gMDSC proportion, as well as with HBV-ACLF prognosis and severity.** (A, B, C) Soluble IDO levels were positively correlated with gMDSC proportion (A), Child-Pugh (B), MELD scores (B), international normalized ratio (C), and total bilirubin (C), but negatively correlated with platelet count (C).
